# Host Cell Antimicrobial Responses against *Helicobacter pylori* Infection: From Biological Aspects to Therapeutic Strategies

**DOI:** 10.3390/ijms231810941

**Published:** 2022-09-19

**Authors:** Judeng Zeng, Chuan Xie, Lin Zhang, Xiaodong Liu, Matthew Tak Vai Chan, William Ka Kei Wu, Huarong Chen

**Affiliations:** 1 Department of Anaesthesia and Intensive Care and Peter Hung Pain Research Institute, The Chinese University of Hong Kong, Hong Kong, China; 2 Shenzhen Research Institute, The Chinese University of Hong Kong, Shenzhen 518172, China; 3 Li Ka Shing Institute of Health Sciences, The Chinese University of Hong Kong, Hong Kong, China; 4 Department of Gastroenterology, The First Affiliated Hospital of Nanchang University, Nanchang 330209, China; 5 State Key Laboratory of Digestive Diseases, The Chinese University of Hong Kong, Hong Kong, China

**Keywords:** *helicobacter pylori*, host cells, antimicrobial responses, antibiotic-resistance

## Abstract

The colonization of *Helicobacter pylori* (*H. pylori*) in human gastric mucosa is highly associated with the occurrence of gastritis, peptic ulcer, and gastric cancer. Antibiotics, including amoxicillin, clarithromycin, furazolidone, levofloxacin, metronidazole, and tetracycline, are commonly used and considered the major treatment regimens for *H. pylori* eradication, which is, however, becoming less effective by the increasing prevalence of *H pylori* resistance. Thus, it is urgent to understand the molecular mechanisms of *H. pylori* pathogenesis and develop alternative therapeutic strategies. In this review, we focus on the virulence factors for *H. pylori* colonization and survival within host gastric mucosa and the host antimicrobial responses against *H. pylori* infection. Moreover, we describe the current treatments for *H. pylori* eradication and provide some insights into new therapeutic strategies for *H. pylori* infection.

## 1. Introduction

Gram-negative, microaerophilic, spiral-shaped *Helicobacter pylori* (*H. pylori*) is commonly identified in the stomach. It has been reported to infect more than a half of the world’s population and can persist life-long without eradication [1,2]. The *H. pylori* infection is thought to occur during childhood within families by the oral-oral or fecal-oral route, with a higher prevalence in developing countries probably due to poor hygiene and crowded conditions [3]. The *H. pylori* infection is a risk factor for chronic gastritis and peptic ulcer and recognized as a class I carcinogen of gastric cancer by the World Health Organization (WHO) [4].

The successful colonization and pathogenesis of *H. pylori* are owing to the action of a variety of bacterial virulence factors. On the one hand, *H. pylori* can generate numerous ureases to neutralize the acidic environment of the stomach lumen. On the other hand, the bacterial flagellar-dependent motility enables *H. pylori* to penetrate the mucus layer toward the gastric epithelium, where the pH is almost neutral [5]. Moreover, a variety of outer membrane proteins (OMPs) of the bacterium serve as adhesins that mediate the adherence of *H. pylori* to the surface of gastric epithelial cells [6]. The colonized *H. pylori* then produce numerous virulence factors, including two well-known cytotoxins-cytotoxic associated gene A (CagA) and vacuolating cytotoxin A (VacA), which can modulate the biological function of gastric epithelial cells and induce the release of proinflammatory cytokines to cause chronic inflammation [7]. For a long time, *H. pylori* was thought to be a non-invasive bacterium thar mainly lived in the mucus layer [8]. However, current evidence has pointed out that a small portion of *H. pylori* can invade and replicate in the intracellular compartments of different cell types [9], causing persistent infection by evading host immune defense and antibiotics [10]. Under this circumstance, host cells have developed numerous antimicrobial responses to fight against invading *H. pylori*, e.g., induction of antimicrobial peptides [11,12], activation of cellular autophagy pathway [13], and increased oxidative stress [14]. 

Eradication of *H. pylori* infection is an effective way to improve or resolve the associated pathology. In clinic, antibiotic-based therapies are usually recommended to treat *H. pylori* infection. However, treatment failure has been increasing in recent years due to poor patient compliance and antibiotic resistance [15]. Therefore, there is an urgent need to develop alternative therapies to fight against *H. pylori* in primary infections or after initial treatment failure. In this review, we aim to describe the interaction between host cells and *H. pylori* and detail the role of host cellular antimicrobial responses against *H. pylori* infection. Moreover, we discuss the current treatments for *H. pylori* infection and highlight key findings of novel alternative therapies, providing some novel insights on developing host cell antimicrobial responses as therapeutic strategies for *H. pylori* eradication.

## 2. Virulence Factors of *H. pylori*

The virulence factors of *H. pylori* are associated with bacterial colonization and the development of gastroduodenal diseases. Here, we briefly introduce some well-known bacterial adhesins and cytotoxins which are involved in host cell-bacterial interaction and pathogenesis. 

### 2.1. Adhesins of H. pylori

The adhesion of *H. pylori* to the gastric epithelium is not only crucial for successful colonization and pathogenesis but also essential for invasion into host cells. Numerous studies have revealed that *H. pylori* expresses a variety of adhesion factors that could bind to related cell surface molecules, such as sugars or proteins. More than 30 *H. pylori* outer membrane proteins (OMPs) have been identified, which play pivotal roles in bacterial attachment to the gastric mucosa. These OMPs could be divided into two groups: Hop (Helicobacter outer membrane proteins) and Hor (Hop-related proteins) subgroups [16,17]. Below, we introduce several members of the Hop-family.

#### 2.1.1. BabA

Blood group antigen-binding adhesin A (BabA) is a major adhesin of *H. pylori*. BabA mediates the adhesion of bacterium to the Lewis b blood group antigen (Le^b^), a major antigen expressed by gastric mucosa [18]. BabA could also bind to salivary mucin MUC5B, gastric mucin MUC5AC, etc. [19,20,21]. It has been reported that BabA-mediated *H. pylori* adherence to Le^b^ on the gastric epithelium promoted type IV secretion system (T4SS) activity, resulting in the production of pro-inflammatory cytokines and other factors that contributed to the development of gastric tumorigenesis [22].

#### 2.1.2. SabA

Sialic acid-binding adherence (SabA), another well-known OMP of *H. pylori*, could bind to sialic acid-modified glycosphingolipids, sialylated Lewis x and Lewis a (sLe^x^ and sLe^a^) [23]. The level of sialylated glycoconjugates is low in healthy population but is induced during gastritis [24]. In addition, sialylated glycoconjugates were increased in *H. pylori*-infected patients but returned to normal after *H. pylori* eradication [25]. Binding of BabA to Le^b^ is thought to be the first step of early *H. pylori* infection, wherein *H. pylori* anchors to the gastric epithelial cell surface. The binding of SabA to sLe^x^ further strengthens the connection [26]. Intriguingly, SabA-mediated binding is weaker than that of BabA, which is probably exploited by *H. pylori* as escape mechanisms from host immune defense responses [27]. Notably, SabA not only functions as an adhesion factor but also serves as a risk factor that is associated with the development of a series of gastroduodenal diseases [28].

#### 2.1.3. OipA

The outer inflammatory protein A (OipA) was initially identified as a *H. pylori* outer membrane protein, which promoted interleukin-8 (IL-8) secretion in a cagPAI-dependent manner [29]. Subsequent studies revealed that OipA could mediate the binding of *H. pylori* to gastric epithelial cells, while the exact host receptor of OipA remains unknown [30]. The phase-variable “on” or “off” status of gene *oipA* of *H. pylori* was found to be associated with the occurrence of gastric cancer [31]. Furthermore, the presence of OipA was able to induce phosphorylation of focal adhesion kinase (FAK) and the downstream extracellular signal-regulated kinases 1 and 2 (Erk1/2) signaling, resulting in cytoskeletal reorganization [32]. 

### 2.2. Cytotoxin-Associated Gene A (CagA) and Type IV Secretion System (T4SS)

Cytotoxin-associated gene A (CagA), the most well-studied virulence factor of *H. pylori*, is a 120–145-kDa immunogenic protein encoded in a 40-kb bacterial genomic DNA region known as cag pathogenicity island (cagPAI) [33]. Presumably, ~31 genes are located in this region and encode proteins of type IV secretion system (T4SS), which is deployed by *H. pylori* to deliver macromolecules into other bacteria or cells [34]. Based on the ability to produce CagA, *H. pylori* can be further classified as CagA-positive and -negative strains [35]. Approximately 30–40% of *H. pylori* strains isolated in Western countries (e.g., America, Australia) are CagA-negative which are less associated with the occurrence of peptic ulcer and gastric carcinogenesis, whereas almost all *H. pylori* strains isolated in East Asian countries (i.e., China, Japan, Korea) are CagA-positive with stronger pathogenicity [36]. 

CagA shows a highly polymorphic Glu-Pro-Ile-Tyr-Ala (EPIYA) repeat region [37]. Accumulating studies have shown that tyrosine phosphorylation in EPIYA motif plays an important role in the cytotoxicity of CagA. *H. pylori* could use T4SS apparatus to translocate CagA into host cells where the tyrosine within EPIYA motif of CagA are phosphorylated by c-Src and c-Abl tyrosine-protein kinases, resulting in disturbed cell signaling pathways and enhanced tumorigenesis [38,39]. In addition, non-phosphorylated CagA was also reported to impair the cellular signal transduction system [40]. It is reported that *H. pylori* CagA could induce epithelial-mesenchymal transition (EMT) in gastric cancer by activating YAP pathway [41]. Another study showed that CagA-positive *H. pylori* promoted DNA damage in gastric cancer via downregulating DNA repair protein Rad51 [42].

### 2.3. Vacuolating Cytotoxin A (VacA)

Vacuolating cytotoxin (VacA) is a secreted toxin encoded by *H. pylori* gene VacA, which is characterized by its ability to form pores and cause vacuolation in cultured eukaryotic cells [43]. VacA is initially produced as a 140-kDa pro-toxin, which is subjected to proteolytic cleavage to yield an active toxin of 88-kDa. Active VacA is secreted extracellularly and undergoes proteolysis to generate two fragments [44] (p. 33 and p. 55). The p. 33 contains a hydrophobic domain and is responsible for pore formation, while p. 55 contains a cell membrane-binding domain and mediates the internalization of VacA [43]. Besides, several VacA-binding receptors were identified at gastric epithelial cell surface, including epidermal growth factor (EGF) [45], receptor protein tyrosine phosphatase alpha/beta (RPTPα/β) [46,47], and low-density lipoprotein receptor-related protein-1 (LRP1) [48]. The internalized VacA exerts a variety of cytotoxic effects, e.g., forming chloride ion channels on late endosomes to induce vacuolation [49], trafficking to mitochondria to induce cytochrome c release and Bax/Bak-dependent apoptosis [50], and inducing autophagy with a reduced autophagic flux [51].

All *H. pylori* strains contain functional but highly variant VacA gene. The different combination of N-terminal “s” region (s1a, s1b, s1c and s2), “m” region (m1, m2) and the intermediate region (i1, i2) determine the pore-forming ability and pathogenicity of VacA [52]. It has been reported that VacA of s1/m1 genotype exhibits the highest vacuolating ability in vitro and is highly associated with the occurrence of gastrointestinal diseases. In contrast, s1/m2 genotype has an intermediate activity while s2/m2 genotype presents almost no cytotoxic activity [53,54]. Interestingly, almost all CagA-positive strains carry s1/m1 VacA while CagA-negative strains are usually s2/m2 genotype [54]. 

## 3. Host Cell Antimicrobial Responses against *H. pylori* Infection

The successful colonization of *H. pylori* results in chronic inflammation and the related pathogenesis in stomach. Meanwhile, host cells also develop numerous antimicrobial responses to defend against *H. pylori* infection. Here, we describe some innate defense strategies exploited by host cells, especially for gastric epithelial cells and professional phagocytic cells, to fight against invading *H. pylori*, including induction of antimicrobial peptides, activation of cellular autophagy pathway, and increased oxidative stress.

### 3.1. Antimicrobial Peptides

Antimicrobial peptides (AMPs), also called host defense peptides (HDPs), are a kind of biologically active small peptides that are widely expressed in almost all living organisms and serve as an important part of the innate immune system to protect the host against a wide spectrum of pathogens [55]. Most AMPs share some common features: (1) The number of amino acids is between 10 and 60, (2) they are cationic and amphipathic and can directly interact with negatively charged bacterial cell membrane to induce pore formation, membrane collapse, and content outflow, (3) they exert immunomodulatory functions, such as induction of pro-inflammatory cytokine release, modulation of the antigen presentation of dendritic cells, and activation of adaptive immune cells [56,57]. Upon *H. pylori* infection, human immune and epithelial cells can produce two major AMPs, cathelicidin/LL-37, and defensins, to protect the host against invading pathogens [58]. 

#### 3.1.1. Cathelicidin/LL-37

As a part of the human innate immune system, cathelicidins are present in a variety of human tissues and organs [59]. Circulating neutrophil, macrophage, as well as epithelial cells of the skin and digestive tract, all express high levels of cathelicidins. In humans, the cathelicidin antimicrobial peptide (*CAMP*) gene encodes the peptide precursor human cationic antimicrobial peptide-18 (hCAP-18), which is then subject to C-terminal cleavage by extracellular serine proteinase-3 to generate active form of LL-37 [60]. LL-37 is the only member of the Cathelicidin family identified in humans. *H. pylori* infection can markedly induce both RNA and protein expression of LL-37 in the gastric epithelium in a T4SS-dependent manner [61]. Mechanically, the mechanistic target of rapamycin complex 1 (mTORC1) was activated in CagA-dependent manner to promote LL-37 expression upon *H. pylori* infection [62]. Activated LL-37 exerted a strong inhibitory effect against *H. pylori* and reduced *H. pylori* colonization in both in vitro gastric epithelial cell line model [63] and in vivo mouse model of gastritis [64]. Due to cationic and amphipathic structural features, LL-37 can rapidly disrupt cell membranes to induce pore formation of the bacteria [65] and further inhibit the formation of bacterial biofilm [66]. In addition to direct killing of microorganisms, LL-37 also exerted an immunomodulatory function by recruiting activated immune cells to infected sites to eliminate the invading bacteria [67,68]. 

#### 3.1.2. Defensins 

Defensins are cysteine-rich cationic AMPs regarded as a part of host innate immune systems to fight against bacterial infection [69]. α-defensin and β-defensin are two major subgroups of defensins in humans, which are widely expressed in immune and epithelial cells [70]. Six members of α-defensin have been identified in humans: Human neutrophil proteins 1 to 4 (HNP1–4) and human defensins 5 and 6 (HD5 and 6), which are secreted by granulocytes and Paneth cells, respectively [71]. *H. pylori* infection was reported to increase HNP1–3 levels in gastric juice and promote the release of active peptides from granulocytes. However, the exact mechanisms of *H. pylori*-induced α-defensin are still unclear [72,73]. On the other hand, more than 50 genes encoding β-defensins (HBDs) have been discovered, which are mainly produced by epithelial tissues. Among them, the roles of HBD1–4 in controlling bacterial infections have been widely studied [71]. HBD1 is constitutively expressed in gastric epithelial cells. The level of HBD1 was moderately increased during *H. pylori* infection [74,75]. Intriguingly, *H. pylori*-induced NLRC4 inflammasome was reported to activate IL-18 to inhibit HBD1 expression in an NF-κB-dependent manner [76]. In contrast, HBD2 expression was markedly elevated upon challenging with cag pathogenicity island positive (cagPAI+) *H. pylori.* Mechanically, internalization of bacterial peptidoglycan caused by cagPAI is recognized by nucleotide-binding oligomerization domain-1 (NOD1), resulting in induction of HBD2 [12]. In addition, HBD3 is induced upon *H. pylori* infection via a TAK1 (transforming growth factor β-activated kinase-1)-EGFR (epidermal growth factor receptor)-p38α axis, which is dependent on the type IV secretion system but independent of CagA or NOD1 [77]. Moreover, CagA-positive strains markedly increased HBD4 expression mediated by p38 [78]. Similar to LL-37, β-defensins exert antimicrobial activity mainly by permeabilizing the bacterial membrane [79]. Furthermore, β-defensins are chemotactic for immune cells, thereby controlling the host immune response to fight against invading *H. pylori* [71]. 

### 3.2. Autophagy Pathway

Autophagy is thought to be the original form of innate immune response of eukaryotic cells against intracellular microorganisms [80]. Besides, previous studies have reported that *H. pylori* could invade the gastric epithelial cells to cause persistent infection [81,82]. The interaction between cellular autophagy and bacterial factors determined the fate of intracellular *H. pylori* [83].

#### 3.2.1. The Definition of Autophagy

“Autophagy” (the Greek word means “self-eating”) refers to cellular machinery that degrades unnecessary and dysfunctional intracellular components through a lysosome-dependent manner [84]. It allows the recycling of cytosolic materials and provides energy to support normal cell activities [85]. Autophagy is induced in response to different kinds of stress, including fasting, nutrient deprivation, infection, and hypoxia, to maintain cellular homeostasis and promote cell survival under harsh conditions. Although autophagy was initially considered a non-selective process, recent studies have pointed out that autophagy could eliminate some unwarned or harmful cytosolic materials, e.g., damaged mitochondria and invaded bacteria, in a selective manner [86]. There are three well-characterized types of autophagy: Macro-autophagy, micro-autophagy, and chaperone-mediated autophagy (CMA). For macro-autophagy, a double-membrane vesicle named autophagosome encloses cytosolic cargos, and then fuses with lysosome to form autolysosome where the cargos are degraded by the hydrolytic enzymes. Macro-autophagy is an important host cell defense mechanism against intracellular *H. pylori* [83].

#### 3.2.2. *H. pylori* Infection and Host Autophagy Pathways

Accumulating evidence suggests that *H. pylori* could invade and multiply in different cell types, such as gastric epithelial cells, macrophage, and dendritic cells [82,87,88]. Terry et al. reported for the first time that attached *H. pylori* could be engulfed by AGS cells through a zipper-like mechanism involving various cellular signal transduction pathways [89]. Autophagy is considered an innate immune response to restrict intracellular bacteria survival. Generally, host cell autophagy is induced upon bacterial infection, as manifested by the formation of autophagosome around invaded bacteria followed by autophagosome-lysosome fusion to degrade bacteria [90]. Terebiznik et al. found that *H. pylori* virulence factor VacA induced autophagy in a manner dependent on the channel-forming activity of VacA, but independent of urease, CagA, or type IV secretion system [91]. Low-density lipoprotein receptor-related protein-1 (LRP1) was identified as a receptor for VacA in gastric epithelial cells to induce autophagy [48]. In addition, intracellular pattern recognition receptor NOD1 could interact with the outer membrane vesicles (OMVs) of *H. pylori* and RIP2 on early endosome to induce cellular autophagy and inflammation, and this effect was independent of VacA [92]. Although autophagy acts as a host innate immune response to eliminate invaded bacteria, *H. pylori* could exploit autophagy and hijack immature autophagosomes to generate a protected reservoir [91,93]. Although acute exposure to VacA induced autophagy in host cells, prolonged exposure to VacA impaired the autophagic flux mainly due to the deficiency of Cathepsin D (CTSD), a principal lysosomal protease in autophagosomes [94]. Capurro et al. further revealed that VacA inhibited the activity of lysosomal calcium channel TRPML1, resulting in disrupted endolysosomal trafficking and decreased mature cathepsin D levels, thus generating an intracellular niche for *H. pylori* [95].

### 3.3. Oxidative and Nitrosative Stress

Colonization of *H. pylori* in gastric epithelium could induce chronic inflammatory response and recruit phagocytic cells, such as macrophages, neutrophils, and inflammatory monocytes. In phagocytes, NADPH oxidase (NOX) and inducible nitric oxide synthase (iNOS), respectively responsible for the generation of superoxide (O_2_^−^) and nitric oxide (NO), are induced to defend against invading pathogens. O_2_^−^ and its derivatives, known as reactive oxygen species (ROS), as well as NO and its derived intermediates, called reactive nitrogen species (RNS), play important roles in host antimicrobial response [96]. In addition to phagocytic cells, gastric epithelial cells also induce NOX and iNOS upon *H. pylori* infection, although how these changes contribute to *H. pylori*-mediated pathogenesis is still controversial [97,98,99].

#### 3.3.1. ROS

The NOXs, which are widely expressed in most mammalian tissues, are responsible for the generation of ROS. *H. pylori* lipopolysaccharide (LPS) was reported to activate the transcription of NOX1 in guinea pig gastric mucosal cells [100]. In addition, phagocyte NOX2 plays a major role in producing ROS and exerts antibacterial effects. Activated phagocytes release ROS both into extracellular space and phagosomal lumens, depending on the cellular location of NOX2 [101,102,103]. NOX2-mediated generation of superoxide (O_2_^−^) and hydrogen peroxide (H_2_O_2_) could directly damage bacterial DNA, lipids, amino acid resides, and Fe-S clusters [101]. Although *H. pylori* induce ROS both in phagocytic cells and gastric epithelial cells, they could express superoxide dismutase (SOD) and catalase to protect themselves [104,105].

#### 3.3.2. RNS

The iNOS are another key enzyme expressed by host immune cells to convert L-arginine to NO through an oxidoreductase reaction [106]. NO could react with superoxide (O_2_^−^) to form oxidant peroxynitrite (ONOO^−^), which exhibits a more powerful effect to kill pathogens [107]. *H. pylori* infection was reported to markedly induce iNOS expression in macrophage and gastric tissues through activating NF-κB signaling [7]. However, Gobert et al. found that *H. pylori* arginase could consume L-arginine, the substrate for iNOS, to inhibit NO production of activated host immune cells [108]. In agreement, Nuruddeen et al. reported that Arginase II of *H. pylori* inhibited iNOS translation in macrophages to restrict host defense [109].

## 4. Current Treatments and Novel Therapeutic Strategies for *H. pylori* Infection

The *H. pylori* infection is highly associated with the occurrence of related gastric disorders. It is now generally accepted that all *H. pylori* infections should be eradicated unless there are compelling reasons. Different guidelines for the management of *H. pylori*-related diseases have been published in many regions. However, the challenge remains huge given the increasing resistance of *H. pylori* to current antibiotic-based treatments. We describe below some novel therapeutic strategies which shed light on the future regimens for *H. pylori* eradication [110,111].

### 4.1. Current Treatments for H. pylori Infection

Antibiotic-based treatments are commonly used to eradicate *H. pylori* infection. It has been shown that a variety of antibiotics, including clarithromycin (CLR), amoxicillin (AMX), and metronidazole (MTZ), could effectively kill *H. pylori* in in vitro environment. However, almost the antibiotics can only work effectively in neutral or near neutral conditions, in contrast to the highly acidic environment of *H. pylori*-resided human stomach. This makes the delivery of active antibiotics more difficult. To solve this problem, a proton pump inhibitor (PPI) is taken together to inhibit the acid secretion by the stomach [111]. More importantly, it is reported that *H. pylori* replicates when stomach pH is larger than 6 and becomes more susceptible to the antibiotic treatment [112]. 

According to the latest version (2017) of the American College of Gastroenterology (ACG) clinical guideline, in the region with low clarithromycin resistance (<15%) rate, a 14-day course of triple therapy containing PPI, CLR, and AMX (or MTZ) is recommended. Patients with penicillin allergy should receive a 10- to 14-day course of bismuth quadruple therapy comprising PPI, bismuth, tetracycline, and a nitroimidazoles. Given the increasing prevalence of CLR resistance worldwide, alternative regimens have been proposed. One of the alternative regimens is 10- to 14-day sequential therapy with PPI and AMX for 5–7 days, followed by PPI, CLR and nitroimidazoles for the next 5–7 days. Another alternative regimen is non-bismuth quadruple (concomitant) therapy containing PPI, CLR, AMX, and nitroimidazoles. Besides, other regimens, including hybrid therapy (a combination of sequential and concomitant therapies) and levofloxacin-based therapy, were also proved to be effective in treating *H. pylori* infection [113,114]. Although numerous therapeutic regimens have been proposed in different guidelines, the success of eradication still depends on many details, which include but are not limited to the choice of treatment options, the duration of treatment, bacterial susceptibility to certain antibiotics, and the patient compliance with the treatment [111].

Notably, recent studies have identified that the addition of probiotics in standard antibiotic therapy could enhance the eradication rate of *H. pylori*, while alleviating the antibiotic-associated adverse effects [115,116]. A meta-analysis found that the supplement of *Bacillus mesentericus* + *Clostridium butyricum* + *Streptococcus faecalis* in a standard triple therapy showed optimal efficacy and safety to enhance the therapeutic effect [117]. Moreover, a great effort was also made to develop a vaccine against *H. pylori* infection. However, no effective vaccines have been applied in clinical practice so far, which is probably due to the high adaptability of *H. pylori* to human immune response and the high genetic diversity of this bacterium [118,119]. 

### 4.2. Novel Therapeutic Strategies for H. pylori Infection

The prevalence of *H pylori* resistance to antibiotics is increasing rapidly worldwide, representing the main challenge in *H. pylori* infection treatment [15,120]. In addition, the presence of intracellular *H. pylori* in gastric biopsies of patients likely confers resistance of *H. pylori* to antibiotics, acid environments, and host immune responses [83,121] and is associated with the failure of first-line triple therapy [122]. Development of non-antibiotic regimens is necessary to eradicate both extracellular and intracellular *H. pylori*. Below, we describe some novel strategies based on modulation of host-bacteria interaction and host antimicrobial responses.

#### 4.2.1. Anti-Adhesive Therapy

Adhesion is a prerequisite for *H. pylori* colonization, invasion, and pathogenesis. Therefore, anti-adhesion therapy is proposed as a novel strategy to control *H. pylori* infection [123]. *H. pylori* could attach to host cells via bacterial adhesin SabA interacting with sialic acid on the cell surface [24]. Numerous sialic acid-containing compounds, such as 3′ Sialyllactose sodium salt, Lactoferrin, and fat globule membrane fractions, have been identified, which effectively inhibit bacterial adhesion. Moreover, sialic acid-based delivery systems have been developed to enhance the eradication rates of *H. pylori* by prolonging drug residence time and increasing drug concentration at infection sites [124]. In addition to sialic acid, Zhang et al. used polymeric nanoparticles coated with outer membrane vesicles of *H. pylori* (OM-NPs) to mimic intact bacteria and found that OM-NPs could compete with bacteria for the cell surface binding sites [125]. Moreover, some food-derived compounds, like Cranberry extracts (proanthocyanidins) and marine algae-derived polysaccharides, also showed an inhibitory effect against bacterial adhesion in gastric mucosa and could be used in the treatment of *H. pylori* infection [126,127,128].

#### 4.2.2. Induction of Antimicrobial Peptides

Human cathelicidin LL-37 peptide and defensins play a pivotal role in the innate defense responses against *H. pylori* infection [63,64,71]. Induction of these antimicrobial peptides could be promising for *H. pylori* treatment. Zhou et al. found that Vitamin D3 (VitD3) could upregulate *CAMP* expression and inhibit *H. pylori* infection in mice, implying that the supplement of VitD3 might help control *H. pylori* infection. Mechanistically, VitD3 bound to vitamin D receptor (VDR) and facilitated its transport to the nucleus. The VitD3/VDR complex could bind to the promoter region of *CAMP* to promote its expression, leading to an enhanced antibacterial effect [129]. Besides, Schauber et al. showed that the use of sodium butyrate, a short chain fatty acids histone-deacetylase inhibitor (HDACi), or Trichostatin (TSA), another HDACi, could increase the acetylation level of histone H3 and H4 accompanied with increased LL-37 expression [130]. Given that HDACi also induced β-defensins expression, HDACi treatment is a novel and attractive strategy for *H. pylori* eradication [131].

#### 4.2.3. Targeting Autophagy Pathway

*H. pylori* can be considered a facultative intracellular bacterium because it can invade host cells and survival in immature autophagosomes [132]. The internalization of *H. pylori* in host cells plays a pivotal role in bacterial virulence and persistence and protects the bacteria from antibiotics [82,133]. *H. pylori* virulence factor VacA could impair lysosomal function by targeting lysosomal Ca^2+^ channel TRPML1 protein to disrupt endolysosomal trafficking, thereby creating an intracellular reservoir [95]. Activation of the cellular autophagy pathway could be promising for eliminating intracellular *H. pylori*. Hu et al. reported that VitD3 treatment promoted the nuclear translocation of PDIA3/STAT3 complex to induce TRPML3 transcription, leading to activated lysosomal function in normal gastric epithelial cells to kill *H. pylori*. Notably, this process is independent of LL-37 activation [134]. Meanwhile, Jones et al. found that TRMPL1 small-molecule agonist ML-SA1 restored lysosomal function of host cells to eradicate *H. pylori* [95]. Therefore, restoring lysosome function is a promising strategy to control intracellular *H. pylori*.

#### 4.2.4. Modulation of Oxidative Stress

The *H. pylori* infection in gastric mucosa recruits immune cells, especially neutrophils, which could release reactive oxygen species (ROS) and reactive nitrogen species (RNS) to kill bacteria [135]. However, persistent production of oxidative stress during chronic *H. pylori* infection could contribute to gastric carcinogenesis [135]. Even worse, *H. pylori* have evolved a variety of strategies to resist oxidative stress [14]. Therefore, it is important to develop therapeutic strategies to enhance the production of ROS and RNS in the region of *H. pylori* infection while avoiding damaging normal tissues. In this regard, Tan et al. synthesized a graphitic nanozyme with pH-responsive oxidase-like activities, which could only catalyze the production of ROS in gastric acidic conditions but not in neutral intestinal environments. Besides, the synthesized nanozyme contains a hydrophobic alkyl tail to enable specific targeting of *H. pylori* and generating of high-concentration ROS only around the bacterial membranes [136]. Another strategy is to abrogate the antioxidant defense of *H. pylori*. HP1021 orphan response regulator was reported to be a redox switch of *H. pylori* to control bacterial response to oxidative stress. Targeting HP1021 could abolish the induction of oxidative stress-related gene expressions in *H. pylori*. Thus, HP1021 is a potential therapeutic target in the treatment of *H. pylori* infection [137]. 

## 5. Conclusions and Future Perspectives

**Figure 1:**
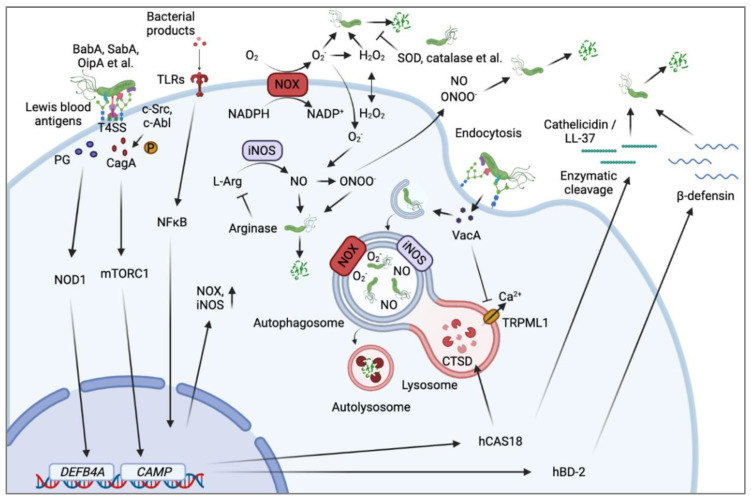
*H. pylori* infection and host antimicrobial response. *H. pylori* attach to host cells surface via bacterial outer membrane proteins (OMPs such as BabA, SabA, OipA, et al.) interacting with ligands (Lewis et al. blood group antigen) on cell surface. Once attached, the bacterial type IV secretion system (T4SS) delivers a variety of virulence factors into host cell. The translocated CagA is phosphorylated by c-Src and c-Abl on the inner face of plasma membrane, leading to the change of numerous cellular signaling pathways. Specifically, CagA can activate mTORC1 to enhance cathelicidin/LL-37 expression. Meanwhile, T4SS-dependent internalization of bacterial peptidoglycan (PG) can activate NOD1 pathway to induce hBD-2/β-defensin expression. The attached bacteria induce cellular endocytosis wherein they are surrounded by the double-membrane structure autophagosome. The autophagosome would then fuse with lysosome to form autolysosome, resulting in bacteria degradation by lysosomal hydrolytic enzymes. However, *H. pylori* could secrete cytotoxin VacA to impair autophagic flux and lysosomal function by inhibiting TRPML1-mediated outflow of Ca2+ on lysosome and suppressing the activity of cathepsin D (CTSD), respectively. Bacterial products can bind to TLRs (Toll-like receptors) to activate NF-κB signaling, resulting in induction of NOX and iNOS and subsequent ROS and RNS generation to kill *H. pylori*. The autophagosome membrane-located NOX and iNOS are also involved in the clearance of intracellular *H. pylori*. Nevertheless, *H. pylori* could produce SOD and catalase to attenuate ROS damage. Moreover, the release of bacterial arginase can eliminate RNS to protect *H. pylori*.

The colonization of *H. pylori* in human gastric mucosa is the main cause of gastritis, peptic ulcers, and gastric cancer [138]. Approximately 20% of *H. pylori* could attach to the gastric epithelial cell surface through the interaction of bacterial outer membrane proteins, e.g., OMPs, BabA, SabA and OipA, with corresponding ligands on host cell membranes [30]. Bacterial adhesion is the prerequisite for *H. pylori* invasion into cells, which may be responsible for immune evasion and treatment failure of *H. pylori* infection [8]. Accordingly, the innate defense system in the body has been developed to fight against invading *H. pylori* through producing antimicrobial peptides (LL-37 and defensins), triggering autophagic degradation of bacteria, and releasing ROS and RNS. Nevertheless, *H. pylori* has also evolved a series of defense systems for their survival (Figure 1).

Currently, antibiotic-based therapies are the major regimens for *H. pylori* eradication. However, treatment failure is increasing because of antibiotic resistance. To avoid antibiotic abuse, development of non-antibiotic therapeutic strategies is urgent. Typing the bacterial susceptibility to antibiotics and determining OMPS and virulence factors would help in further individualization of the treatment. Besides, modulation of host antimicrobial responses is promising to fight against invading *H. pylori*. Recent studies reported that administration of VitD and HDACi was shown to increase antimicrobial peptide levels [131,139]. Notably, antimicrobial peptide LL-37 could not only kill extracellular bacteria, but also facilitate the clearance of invaded pathogen [140]. In addition to LL-37, autophagy-activating agents also effectively inhibited intracellular bacteria load [141]. Thus, activation of the host defense through autophagy against *H. pylori* infection is another alternative therapeutic strategy. Lastly, therapeutic strategies to enhance ROS and RNS level in the region of *H. pylori* infection while abrogating its antioxidant response is a new weapon to kill invaded *H. pylori* [136,137]. In sum, modulation of host antimicrobial responses is promising for controlling *H. pylori* infection and could be used together to enhance the efficacy of antibiotics-based regimens or used as alternative regimens when current first-line treatment has failed in the future. However, preclinical and clinical studies are required to assess their efficacy and adverse effects, and the cost and compliance of patients should also be considered.

## Data Availability

Not applicable.

## References

[1] Kusters G.J., van Vliet A.H., Kuipers E.J. (2006). Pathogenesis of helicobacter pylori infection. Clin. Microbiol. Rev..

[2] Sabbagh P., Mohammadnia-Afrouzi M., Javanian M., Babazadeh A., Koppolu V., Vasigala V.R., Nouri H.R., Ebrahimpour S. (2019). Diagnostic methods for helicobacter pylori infection: Ideals, options, and limitations. Eur. J. Clin. Microbiol. Infect. Dis..

[3] Vale F.F., Vitor J.M. (2010). Transmission pathway of helicobacter pylori: Does food play a role in rural and urban areas?. Int. J. Food Microbiol..

[4] Conteduca V., Sansonno D., Lauletta G., Russi S., Ingravallo G., Dammacco F.H. (2013). Pylori infection and gastric cancer: State of the art (review). Int. J. Oncol..

[5] Yang I., Nell S., Suerbaum S. (2013). Survival in hostile territory: The microbiota of the stomach. FEMS Microbiol. Rev..

[6] Oleastro M., Menard A. (2013). The role of helicobacter pylori outer membrane proteins in adherence and pathogenesis. Biology.

[7] Lamb A., Chen L.F. (2013). Role of the helicobacter pylori-induced inflammatory response in the development of gastric cancer. J. Cell Biochem..

[8] Huang Y., Wang Q.L., Cheng D.D., Xu W.T., Lu N.H. (2016). Adhesion and invasion of gastric mucosa epithelial cells by helicobacter pylori. Front. Cell Infect. Microbiol..

[9] Ricci V., Romano M., Boquet P. (2011). Molecular cross-talk between helicobacter pylori and human gastric mucosa. World J. Gastroenterol..

[10] Lina T.T., Alzahrani S., Gonzalez J., Pinchuk I.V., Beswick E.J., Reyes V.E. (2014). Immune evasion strategies used by helicobacter pylori. World J. Gastroenterol..

[11] Moretta A., Scieuzo C., Petrone A.M., Salvia R., Manniello M.D., Franco A., Lucchetti D., Vassallo A., Vogel H., Sgambato A. (2021). Antimicrobial peptides: A new hope in biomedical and pharmaceutical fields. Front. Cell Infect. Microbiol..

[12] Grubman A., Kaparakis M., Viala J., Allison C., Badea L., Karrar A., Boneca I.G., le Bourhis L., Reeve S., Smith I.A. (2010). The innate immune molecule, nod1, regulates direct killing of helicobacter pylori by antimicrobial peptides. Cell Microbiol..

[13] Raju D., Hussey S., Jones N.L. (2012). Crohn disease atg16l1 polymorphism increases susceptibility to infection with helicobacter pylori in humans. Autophagy.

[14] Flint A., Stintzi A., Saraiva L.M. (2016). Oxidative and nitrosative stress defences of helicobacter and campylobacter species that counteract mammalian immunity. FEMS Microbiol. Rev..

[15] Tshibangu-Kabamba E., Yamaoka Y. (2021). Helicobacter pylori infection and antibiotic resistance—from biology to clinical implications. Nat. Rev. Gastroenterol. Hepatol..

[16] Backert S., Clyne M., Tegtmeyer N. (2011). Molecular mechanisms of gastric epithelial cell adhesion and injection of caga by helicobacter pylori. Cell Commun. Signal.

[17] Matos R., Amorim I., Magalhaes A., Haesebrouck F., Gartner F., Reis C.A. (2021). Adhesion of helicobacter species to the human gastric mucosa: A deep look into glycans role. Front. Mol. Biosci..

[18] Hage N., Howard T., Phillips C., Brassington C., Overman R., Debreczeni J., Gellert P., Stolnik S., Winkler G.S., Falcone F.H. (2015). Structural basis of lewis(b) antigen binding by the helicobacter pylori adhesin baba. Sci. Adv..

[19] Ansari S., Yamaoka Y. (2017). Helicobacter pylori baba in adaptation for gastric colonization. World J. Gastroenterol..

[20] Kenny D.T., Skoog E.C., Linden S.K., Struwe W.B., Rudd P.M., Karlsson N.G. (2012). Presence of terminal n-acetylgalactosaminebeta1-4n-acetylglucosamine residues on o-linked oligosaccharides from gastric muc5ac: Involvement in helicobacter pylori colonization?. Glycobiology.

[21] Walz A., Odenbreit S., Mahdavi J., Boren T., Ruhl S. (2005). Identification and characterization of binding properties of helicobacter pylori by glycoconjugate arrays. Glycobiology.

[22] Ishijima N., Suzuki M., Ashida H., Ichikawa Y., Kanegae Y., Saito I., Boren T., Haas R., Sasakawa C., Mimuro H. (2011). Baba-mediated adherence is a potentiator of the helicobacter pylori type iv secretion system activity. J. Biol. Chem..

[23] Posselt G., Backert S., Wessler S. (2013). The functional interplay of helicobacter pylori factors with gastric epithelial cells induces a multi-step process in pathogenesis. Cell Commun. Signal.

[24] Benktander J., Barone A., Johansson M.M., Teneberg S. (2018). Helicobacter pylori saba binding gangliosides of human stomach. Virulence.

[25] Ota H., Nakayama J., Momose M., Hayama M., Akamatsu T., Katsuyama T., Graham D.Y., Genta R.M. (1998). Helicobacter pylori infection produces reversible glycosylation changes to gastric mucins. Virchows Arch..

[26] Doohan D., Rezkitha Y.A.A., Waskito L.A., Yamaoka Y., Miftahussurur M. (2021). Helicobacter pylori baba-saba key roles in the adherence phase: The synergic mechanism for successful colonization and disease development. Toxins.

[27] Mahdavi J., Sonden B., Hurtig M., Olfat F.O., Forsberg L., Roche N., Angstrom J., Larsson T., Teneberg S., Karlsson K.A. (2002). Helicobacter pylori saba adhesin in persistent infection and chronic inflammation. Science.

[28] Yamaoka Y., Ojo O., Fujimoto S., Odenbreit S., Haas R., Gutierrez O., El-Zimaity H.M., Reddy R., Arnqvist A., Graham D.Y. (2006). Helicobacter pylori outer membrane proteins and gastroduodenal disease. Gut.

[29] Horridge D.N., Begley A.A., Kim J., Aravindan N., Fan K., Forsyth M.H. (2017). Outer inflammatory protein a (oipa) of helicobacter pylori is regulated by host cell contact and mediates caga translocation and interleukin-8 response only in the presence of a functional cag pathogenicity island type iv secretion system. Pathog. Dis..

[30] Alzahrani S., Lina T.T., Gonzalez J., Pinchuk I.V., Beswick E.J., Reyes V.E. (2014). Effect of helicobacter pylori on gastric epithelial cells. World J. Gastroenterol..

[31] Braga L., Batista M.H.R., de Azevedo O.G.R., Costa K.C.d., Gomes A.D., Rocha G.A., Queiroz D.M.M. (2019). Oipa “on” status of helicobacter pylori is associated with gastric cancer in north-eastern brazil. BMC Cancer.

[32] Tabassam F.H., Graham D.Y., Yamaoka Y. (2008). Oipa plays a role in helicobacter pylori-induced focal adhesion kinase activation and cytoskeletal re-organization. Cell Microbiol..

[33] Ansari S., Yamaoka Y. (2020). Helicobacter pylori virulence factor cytotoxin-associated gene a (caga)-mediated gastric pathogenicity. Int. J. Mol. Sci..

[34] Cover T.L., Lacy D.B., Ohi M.D. (2020). The helicobacter pylori cag type iv secretion system. Trends Microbiol..

[35] Schneider N., Krishna U., Romero-Gallo J., Israel D.A., Piazuelo M.B., Camargo M.C., Sicinschi L.A., Schneider B.G., Correa P., Peek R.M. (2009). Role of helicobacter pylori caga molecular variations in induction of host phenotypes with carcinogenic potential. J. Infect. Dis..

[36] Chang W.L., Yeh Y.C., Sheu B.S. (2018). The impacts of h. Pylori virulence factors on the development of gastroduodenal diseases. J. Biomed. Sci..

[37] Hatakeyama M. (2017). Structure and function of helicobacter pylori caga, the first-identified bacterial protein involved in human cancer. Proc. Jpn. Acad. Ser. B Phys. Biol. Sci..

[38] Takahashi-Kanemitsu A., Knight C.T., Hatakeyama M. (2020). Molecular anatomy and pathogenic actions of helicobacter pylori caga that underpin gastric carcinogenesis. Cell Mol. Immunol..

[39] Cover T.L. (2016). Helicobacter pylori diversity and gastric cancer risk. mBio.

[40] Backert S., Tegtmeyer N., Selbach M. (2010). The versatility of helicobacter pylori caga effector protein functions: The master key hypothesis. Helicobacter.

[41] Li N., Feng Y., Hu Y., He C., Xie C., Ouyang Y., Artim S.C., Huang D., Zhu Y., Luo Z. (2018). Helicobacter pylori caga promotes epithelial mesenchymal transition in gastric carcinogenesis via triggering oncogenic yap pathway. J. Exp. Clin. Cancer Res..

[42] Xie C., Li N., Wang H., He C., Hu Y., Peng C., Ouyang Y., Wang D., Xie Y., Chen J. (2020). Inhibition of autophagy aggravates DNA damage response and gastric tumorigenesis via rad51 ubiquitination in response to h. Pylori infection. Gut Microbes.

[43] Ansari S., Yamaoka Y. (2019). Helicobacter pylori virulence factors exploiting gastric colonization and its pathogenicity. Toxins.

[44] Ricci V. (2016). Relationship between vaca toxin and host cell autophagy in helicobacter pylori infection of the human stomach: A few answers, many questions. Toxins.

[45] Seto K., Hayashi-Kuwabara Y., Yoneta T., Suda H., Tamaki H. (1998). Vacuolation induced by cytotoxin from helicobacter pylori is mediated by the egf receptor in hela cells. FEBS Lett..

[46] Yahiro K., Wada A., Nakayama M., Kimura T., Ogushi K., Niidome T., Aoyagi H., Yoshino K., Yonezawa K., Moss J. (2003). Protein-tyrosine phosphatase alpha, rptp alpha, is a helicobacter pylori vaca receptor. J. Biol. Chem..

[47] Yahiro K., Niidome T., Kimura M., Hatakeyama T., Aoyagi H., Kurazono H., Imagawa K., Wada A., Moss J., Hirayama T. (1999). Activation of helicobacter pylori vaca toxin by alkaline or acid conditions increases its binding to a 250-kda receptor protein-tyrosine phosphatase beta. J. Biol. Chem..

[48] Yahiro K., Satoh M., Nakano M., Hisatsune J., Isomoto H., Sap J., Suzuki H., Nomura F., Noda M., Moss J. (2012). Low-density lipoprotein receptor-related protein-1 (lrp1) mediates autophagy and apoptosis caused by helicobacter pylori vaca. J. Biol. Chem..

[49] Palframan S.L., Kwok T., Gabriel K. (2012). Vacuolating cytotoxin a (vaca), a key toxin for helicobacter pylori pathogenesis. Front. Cell Infect. Microbiol..

[50] Yamasaki E., Wada A., Kumatori A., Nakagawa I., Funao J., Nakayama M., Hisatsune J., Kimura M., Moss J., Hirayama T. (2006). Helicobacter pylori vacuolating cytotoxin induces activation of the proapoptotic proteins bax and bak, leading to cytochrome c release and cell death, independent of vacuolation. J. Biol. Chem..

[51] Greenfield L.K., Jones N.L. (2013). Modulation of autophagy by helicobacter pylori and its role in gastric carcinogenesis. Trends Microbiol..

[52] Trang T.T.H., Binh T.T., Yamaoka Y. (2016). Relationship between vaca types and development of gastroduodenal diseases. Toxins.

[53] Aftab H., Miftahussurur M., Subsomwong P., Ahmed F., Khan A.K.A., Matsumoto T., Suzuki R., Yamaoka Y. (2017). Two populations of less-virulent helicobacter pylori genotypes in bangladesh. PLoS ONE.

[54] Sahara S., Sugimoto M., Vilaichone R.K., Mahachai V., Miyajima H., Furuta T., Yamaoka Y. (2012). Role of helicobacter pylori caga epiya motif and vaca genotypes for the development of gastrointestinal diseases in southeast asian countries: A meta-analysis. BMC Infect. Dis..

[55] Drayton M., Deisinger J.P., Ludwig K.C., Raheem N., Muller A., Schneider T., Straus S.K. (2021). Host defense peptides: Dual antimicrobial and immunomodulatory action. Int. J. Mol. Sci..

[56] Huan Y., Kong Q., Mou H., Yi H. (2020). Antimicrobial peptides: Classification, design, application and research progress in multiple fields. Front. Microbiol..

[57] Neshani A., Zare H., Eidgahi M.R.A., Chichaklu A.H., Movaqar A., Ghazvini K. (2019). Review of antimicrobial peptides with anti-helicobacter pylori activity. Helicobacter.

[58] Wang G. (2014). Human antimicrobial peptides and proteins. Pharmaceuticals.

[59] Kosciuczuk E.M., Lisowski P., Jarczak J., Strzalkowska N., Jozwik A., Horbanczuk J., Krzyzewski J., Zwierzchowski L., Bagnicka E. (2012). Cathelicidins: Family of antimicrobial peptides. A review. Mol. Biol. Rep..

[60] Alford M.A., Baquir B., Santana F.L., Haney E.F., Hancock R.E.W. (2020). Cathelicidin host defense peptides and inflammatory signaling: Striking a balance. Front. Microbiol..

[61] Hase K., Murakami M., Iimura M., Cole S.P., Horibe Y., Ohtake T., Obonyo M., Gallo R.L., Eckmann L., Kagnoff M.F. (2003). Expression of ll-37 by human gastric epithelial cells as a potential host defense mechanism against helicobacter pylori. Gastroenterology.

[62] Feng G.J., Chen Y., Li K. (2020). Helicobacter pylori promote inflammation and host defense through the caga-dependent activation of mtorc1. J. Cell Physiol..

[63] Zhang L., Wu W.K., Gallo R.L., Fang E.F., Hu W., Ling T.K., Shen J., Chan R.L., Lu L., Luo X.M. (2016). Critical role of antimicrobial peptide cathelicidin for controlling helicobacter pylori survival and infection. J. Immunol..

[64] Zhang L., Yu J., Wong C.C., Ling T.K., Li Z.J., Chan K.M., Ren S.X., Shen J., Chan R.L., Lee C.C. (2013). Cathelicidin protects against helicobacter pylori colonization and the associated gastritis in mice. Gene Ther..

[65] Bahar A.A., Ren D. (2013). Antimicrobial peptides. Pharmaceuticals.

[66] Dosler S., Karaaslan E. (2014). Inhibition and destruction of pseudomonas aeruginosa biofilms by antibiotics and antimicrobial peptides. Peptides.

[67] Kumar P., Kizhakkedathu J.N., Straus S.K. (2018). Antimicrobial peptides: Diversity, mechanism of action and strategies to improve the activity and biocompatibility in vivo. Biomolecules.

[68] Hilchie A.L., Wuerth K., Hancock R.E. (2013). Immune modulation by multifaceted cationic host defense (antimicrobial) peptides. Nat. Chem. Biol..

[69] Solanki S.S., Singh P., Kashyap P., Sansi M.S., Ali S.A. (2021). Promising role of defensins peptides as therapeutics to combat against viral infection. Microb. Pathog..

[70] Xu D., Lu W. (2020). Defensins: A double-edged sword in host immunity. Front. Immunol..

[71] Pero R., Coretti L., Nigro E., Lembo F., Laneri S., Lombardo B., Daniele A., Scudiero O. (2017). Beta-defensins in the fight against helicobacter pylori. Molecules.

[72] Kocsis A.K., Ocsovszky I., Tiszlavicz L., Tiszlavicz Z., Mandi Y. (2009). Helicobacter pylori induces the release of alpha-defensin by human granulocytes. Inflamm. Res..

[73] Isomoto H., Mukae H., Ishimoto H., Date Y., Nishi Y., Inoue K., Wada A., Hirayama T., Nakazato M., Kohno S. (2004). Elevated concentrations of alpha-defensins in gastric juice of patients with helicobacter pylori infection. Am. J. Gastroenterol..

[74] O’Neil D.A., Cole S.P., Martin-Porter E., Housley M.P., Liu L., Ganz T., Kagnoff M.F. (2000). Regulation of human beta-defensins by gastric epithelial cells in response to infection with helicobacter pylori or stimulation with interleukin-1. Infect. Immun..

[75] Bajaj-Elliott M., Fedeli P., Smith G.V., Domizio P., Maher L., Ali R.S., Quinn A.G., Farthing M.J. (2002). Modulation of host antimicrobial peptide (beta-defensins 1 and 2) expression during gastritis. Gut.

[76] Semper R.P., Vieth M., Gerhard M., Mejias-Luque R. (2019). Helicobacter pylori exploits the nlrc4 inflammasome to dampen host defenses. J. Immunol..

[77] Muhammad J.S., Zaidi S.F., Zhou Y., Sakurai H., Sugiyam T.a. (2016). Novel epidermal growth factor receptor pathway mediates release of human beta-defensin 3 from helicobacter pylori-infected gastric epithelial cells. Pathog. Dis..

[78] Otte J.M., Neumann H.M., Brand S., Schrader H., Schmidt W.E., Schmitz F. (2009). Expression of beta-defensin 4 is increased in human gastritis. Eur. J. Clin. Investig..

[79] Zharkova M.S., Orlov D.S., Golubeva O.Y., Chakchir O.B., Eliseev I.E., Grinchuk T.M., Shamova O.V. (2019). Application of antimicrobial peptides of the innate immune system in combination with conventional antibiotics-a novel way to combat antibiotic resistance?. Front. Cell Infect. Microbiol..

[80] Deretic V., Saitoh T., Akira S. (2013). Autophagy in infection, inflammation and immunity. Nat. Rev. Immunol..

[81] Dubois A., Boren T. (2007). Helicobacter pylori is invasive and it may be a facultative intracellular organism. Cell Microbiol..

[82] Bjorkholm B., Zhukhovitsky V., Lofman C., Hulten K., Enroth H., Block M., Rigo R., Falk P., Engstrand L. (2000). Helicobacter pylori entry into human gastric epithelial cells: A potential determinant of virulence, persistence, and treatment failures. Helicobacter.

[83] Deen N.S., Huang S.J., Gong L., Kwok T., Devenish R.J. (2013). The impact of autophagic processes on the intracellular fate of helicobacter pylori: More tricks from an enigmatic pathogen?. Autophagy.

[84] De Duve C., Pressman B.C., Gianetto R., Wattiaux R., Appelmans F. (1955). Tissue fractionation studies. 6. Intracellular distribution patterns of enzymes in rat-liver tissue. Biochem. J..

[85] Chang N.C. (2020). Autophagy and stem cells: Self-eating for self-renewal. Front. Cell Dev. Biol..

[86] Dikic I., Elazar Z. (2018). Mechanism and medical implications of mammalian autophagy. Nat. Rev. Mol. Cell Biol..

[87] Wang Y.H., Wu J.J., Lei H.Y. (2009). When helicobacter pylori invades and replicates in the cells. Autophagy.

[88] Ko G.H., Kang S.M., Kim Y.K., Lee J.H., Park C.K., Youn H.S., Baik S.C., Cho M.J., Lee W.K., Rhee K.H. (1999). Invasiveness of helicobacter pylori into human gastric mucosa. Helicobacter.

[89] Kwok T., Backert S., Schwarz H., Berger J., Meyer T.F. (2002). Specific entry of helicobacter pylori into cultured gastric epithelial cells via a zipper-like mechanism. Infect. Immun..

[90] Hu W., Chan H., Lu L., Wong K.T., Wong S.H., Li M.X., Xiao Z.G., Cho C.H., Gin T., Chan M.T.V. (2020). Autophagy in intracellular bacterial infection. Semin. Cell Dev. Biol..

[91] Terebiznik M.R., Raju D., Vazquez C.L., Torbricki K., Kulkarni R., Blanke S.R., Yoshimori T., Colombo M.I., Jones N.L. (2009). Effect of helicobacter pylori’s vacuolating cytotoxin on the autophagy pathway in gastric epithelial cells. Autophagy.

[92] Irving A.T., Mimuro H., Kufer T.A., Lo C., Wheeler R., Turner L.J., Thomas B.J., Malosse C., Gantier M.P., Casillas L.N. (2014). The immune receptor nod1 and kinase rip2 interact with bacterial peptidoglycan on early endosomes to promote autophagy and inflammatory signaling. Cell Host Microbe.

[93] Orvedahl A., Levine B. (2009). Eating the enemy within: Autophagy in infectious diseases. Cell Death Differ..

[94] Raju D., Hussey S., Ang M., Terebiznik M.R., Sibony M., Galindo-Mata E., Gupta V., Blanke S.R., Delgado A., Romero-Gallo J. (2012). Vacuolating cytotoxin and variants in atg16l1 that disrupt autophagy promote helicobacter pylori infection in humans. Gastroenterology.

[95] Capurro M.I., Greenfield L.K., Prashar A., Xia S., Abdullah M., Wong H., Zhong X.Z., Bertaux-Skeirik N., Chakrabarti J., Siddiqui I. (2019). Vaca generates a protective intracellular reservoir for helicobacter pylori that is eliminated by activation of the lysosomal calcium channel trpml1. Nat. Microbiol..

[96] Fang F.C. (2004). Antimicrobial reactive oxygen and nitrogen species: Concepts and controversies. Nat. Rev. Microbiol..

[97] Handa O., Naito Y., Yoshikawa T. (2011). Redox biology and gastric carcinogenesis: The role of helicobacter pylori. Redox Rep..

[98] Angrisano T., Lembo F., Peluso S., Keller S., Chiariotti L., Pero R. (2012). Helicobacter pylori regulates inos promoter by histone modifications in human gastric epithelial cells. Med. Microbiol. Immunol..

[99] Echizen K., Horiuchi K., Aoki Y., Yamada Y., Minamoto T., Oshima H., Oshima M. (2019). NF-kappab-induced nox1 activation promotes gastric tumorigenesis through the expansion of sox2-positive epithelial cells. Oncogene.

[100] Kawahara T., Kohjima M., Kuwano Y., Mino H., Teshima-Kondo S., Takeya R., Tsunawaki S., Wada A., Sumimoto H., Rokutan K. (2005). Helicobacter pylori lipopolysaccharide activates rac1 and transcription of nadph oxidase nox1 and its organizer noxo1 in guinea pig gastric mucosal cells. Am. J. Physiol. Cell Physiol..

[101] Slauch J.M. (2011). How does the oxidative burst of macrophages kill bacteria? Still an open question. Mol. Microbiol..

[102] Hurst J.K. (2012). What really happens in the neutrophil phagosome?. Free Radic. Biol. Med..

[103] Holmdahl R., Sareila O., Olsson L.M., Backdahl L., Wing K. (2016). Ncf1 polymorphism reveals oxidative regulation of autoimmune chronic inflammation. Immunol. Rev..

[104] Ramarao N., Gray-Owen S.D., Meyer T.F. (2000). Helicobacter pylori induces but survives the extracellular release of oxygen radicals from professional phagocytes using its catalase activity. Mol. Microbiol..

[105] Seyler R.W., Olson J.W., Maier R.J. (2001). Superoxide dismutase-deficient mutants of helicobacter pylori are hypersensitive to oxidative stress and defective in host colonization. Infect. Immun..

[106] Bogdan C. (2015). Nitric oxide synthase in innate and adaptive immunity: An update. Trends Immunol..

[107] Radi R. (2018). Oxygen radicals, nitric oxide, and peroxynitrite: Redox pathways in molecular medicine. Proc. Natl. Acad. Sci. USA.

[108] Gobert A.P., McGee D.J., Akhtar M., Mendz G.L., Newton J.C., Cheng Y., Mobley H.L., Wilson K.T. (2001). Helicobacter pylori arginase inhibits nitric oxide production by eukaryotic cells: A strategy for bacterial survival. Proc. Natl. Acad. Sci. USA.

[109] Lewis N.D., Asim M., Barry D.P., Singh K., de Sablet T., Boucher J.L., Gobert A.P., Chaturvedi R., Wilson K.T. (2010). Arginase ii restricts host defense to helicobacter pylori by attenuating inducible nitric oxide synthase translation in macrophages. J. Immunol..

[110] Suzuki S., Kusano C., Horii T., Ichijima R., Ikehara H. (2022). The ideal helicobacter pylori treatment for the present and the future. Digestion.

[111] Matsumoto H., Shiotani A., Graham D.Y. (2019). Current and future treatment of helicobacter pylori infections. Adv. Exp. Med. Biol..

[112] Scott D., Weeks D., Melchers K., Sachs G. (1998). The life and death of helicobacter pylori. Gut.

[113] Chey W.D., Leontiadis G.I., Howden C.W., Moss S.F. (2017). Acg clinical guideline: Treatment of helicobacter pylori infection. Am. J. Gastroenterol..

[114] Kamboj A.K., Cotter T.G., Oxentenko A.S. (2017). Helicobacter pylori: The past, present, and future in management. Mayo Clin. Proc..

[115] Ji J., Yang H. (2020). Using probiotics as supplementation for helicobacter pylori antibiotic therapy. Int. J. Mol. Sci..

[116] Homan M., Orel R. (2015). Are probiotics useful in helicobacter pylori eradication?. World J. Gastroenterol..

[117] Wen J., Peng P., Chen P., Zeng L., Pan Q., Wei W., He J. (2017). Probiotics in 14-day triple therapy for asian pediatric patients with helicobacter pylori infection: A network meta-analysis. Oncotarget.

[118] Abadi A.T.B. (2016). Vaccine against helicobacter pylori: Inevitable approach. World J. Gastroenterol..

[119] Agarwal K., Agarwal S. (2008). Helicobacter pylori vaccine: From past to future. Mayo Clin. Proc..

[120] Megraud F. (2004). H pylori antibiotic resistance: Prevalence, importance, and advances in testing. Gut.

[121] Abadi A.T.B. (2017). Strategies used by helicobacter pylori to establish persistent infection. World J. Gastroenterol..

[122] Beer A., Hudler H., Hader M., Kundi M., Hudler S., Tauber V., Schachner H., Gruber S., Hirschl A.M., Kain R. (2021). Apparent intracellular helicobacter pylori detected by immunohistochemistry: The missing link in eradication failure. Clin. Infect. Dis..

[123] Asadi A., Razavi S., Talebi M., Gholami M. (2019). Correction to: A review on anti-adhesion therapies of bacterial diseases. Infection.

[124] Sun X., Zhang S., Ren J., Udenigwe C.C. (2022). Sialic acid-based strategies for the prevention and treatment of helicobacter pylori infection: Emerging trends in food industry. Crit. Rev. Food Sci. Nutr..

[125] Zhang Y., Chen Y., Lo C., Zhuang J., Angsantikul P., Zhang Q., Wei X., Zhou Z., Obonyo M., Fang R.H. (2019). Inhibition of pathogen adhesion by bacterial outer membrane-coated nanoparticles. Angew. Chem. Int. Ed. Engl..

[126] Menchicchi B., Hensel A., Goycoolea F.M. (2015). Polysaccharides as bacterial antiadhesive agents and “smart” constituents for improved drug delivery systems against helicobacter pylori infection. Curr. Pharm. Des..

[127] Besednova N.N., Zaporozhets T.S., Somova L.M., Kuznetsova T.A. (2015). Review: Prospects for the use of extracts and polysaccharides from marine algae to prevent and treat the diseases caused by helicobacter pylori. Helicobacter.

[128] Shmuely H., Ofek I., Weiss E.I., Rones Z., Houri-Haddad Y. (2012). Cranberry components for the therapy of infectious disease. Curr. Opin. Biotechnol..

[129] Zhou A., Li L., Zhao G., Min L., Liu S., Zhu S., Guo Q., Liu C., Zhang S., Li P. (2020). Vitamin d3 inhibits helicobacter pylori infection by activating the vitd3/vdr-camp pathway in mice. Front. Cell Infect. Microbiol..

[130] Schauber J., Iffland K., Frisch S., Kudlich T., Schmausser B., Eck M., Menzel T., Gostner A., Luhrs H., Scheppach W. (2004). Histone-deacetylase inhibitors induce the cathelicidin ll-37 in gastrointestinal cells. Mol. Immunol..

[131] Yedery R.D., Jerse A.E. (2015). Augmentation of cationic antimicrobial peptide production with histone deacetylase inhibitors as a novel epigenetic therapy for bacterial infections. Antibiotics.

[132] Sit W.Y., Chen Y.A., Chen Y.L., Lai C.H., Wang W.C. (2020). Cellular evasion strategies of helicobacter pylori in regulating its intracellular fate. Semin. Cell Dev. Biol..

[133] Wang Y.H., Lv Z.F., Zhong Y., Liu D.S., Chen S.P., Xie Y. (2017). The internalization of helicobacter pylori plays a role in the failure of h. Pylori eradication. Helicobacter.

[134] Hu W., Zhang L., Li M.X., Shen J., Liu X.D., Xiao Z.G., Wu D.L., Ho I.H.T., Wu J.C.Y., Cheung C.K.Y. (2019). Vitamin d3 activates the autolysosomal degradation function against helicobacter pylori through the pdia3 receptor in gastric epithelial cells. Autophagy.

[135] Butcher L.D., den Hartog G., Ernst P.B., Crowe S.E. (2017). Oxidative stress resulting from helicobacter pylori infection contributes to gastric carcinogenesis. Cell Mol. Gastroenterol. Hepatol..

[136] Zhang L., Zhang L., Deng H., Li H., Tang W., Guan L., Qiu Y., Donovan M.J., Chen Z., Tan W. (2021). In vivo activation of ph-responsive oxidase-like graphitic nanozymes for selective killing of helicobacter pylori. Nat. Commun..

[137] Szczepanowski P., Noszka M., Zyla-Uklejewicz D., Pikula F., Nowaczyk-Cieszewska M., Krezel A., Stingl K., Zawilak-Pawlik A. (2021). Hp1021 is a redox switch protein identified in helicobacter pylori. Nucleic Acids Res..

[138] Wroblewski L.E., Peek R.M., Wilson K.T. (2010). Helicobacter pylori and gastric cancer: Factors that modulate disease risk. Clin. Microbiol. Rev..

[139] Gombart A.F. (2009). The vitamin d-antimicrobial peptide pathway and its role in protection against infection. Future Microbiol..

[140] Bosch M., Sanchez-Alvarez M., Fajardo A., Kapetanovic R., Steiner B., Dutra F., Moreira L., Lopez J.A., Campo R., Mari M. (2020). Mammalian lipid droplets are innate immune hubs integrating cell metabolism and host defense. Science.

[141] Kim Y.S., Silwal P., Kim S.Y., Yoshimori T., Jo E.K. (2019). Autophagy-activating strategies to promote innate defense against mycobacteria. Exp. Mol. Med..

